# The Antineoplastic Effect of Heparin on Colorectal Cancer: A Review of the Literature

**DOI:** 10.3390/jcm12227173

**Published:** 2023-11-19

**Authors:** Emmanuel Giannas, Christos Kontovounisios

**Affiliations:** 1 Department of Surgery and Cancer, Imperial College London, London SW7 2BX, UK; eg1818@imperial.ac.uk; 2 Department of General Surgery, Chelsea and Westminster Hospital, London SW10 9NH, UK; 3 Department of Surgery, The Royal Marsden Hospital, London SW3 6JJ, UK

**Keywords:** heparin, LMWH, antineoplastic, anticarcinogenic, colorectal cancer

## Abstract

Heparin and derivatives are commonly used for thrombophylaxis in surgical colorectal cancer (CRC) patients. Recent studies have suggested that, besides its protective effect on the incidence of venous thromboembolism, heparin has an anti-cancer effect. The aim of this review was to explore the literature and report the antineoplastic effect of heparin and derivatives on CRC. MEDLINE and EMBASE databases were searched for relevant articles. Nineteen studies were included (*n* = 19). Fifteen were lab studies conducted in vivo or in vitro on CRC cell lines and/or mice (*n* = 15). Four were in vivo clinical studies (*n* = 4). CRC tumor growth was reduced by 78% in one study, (*p* < 0.01), while tumorigenesis was suppressed in heparin-treated mice in seven studies. A high dose of low molecular weight heparin for extended duration significantly reduced post-operative VEGF, suggesting that such a regime may inhibit tumor angiogenesis and distant metastasis. A randomized trial demonstrated the antineoplastic effect of nadroparin as the 6 month survival in palliative patients increased. Another study has reported that disease-free survival of CRC patients was not affected by a similar tinzaparin regime. The anti-cancer properties of heparin and derivatives are promising, especially in lab studies. Further clinical trials are needed to investigate the anti-cancer benefit of heparin on CRC.

## 1. Introduction

Heparin, and in particular low molecular weight heparin (LMWH), is commonly used for thrombophylaxis in colorectal cancer (CRC) patients undergoing curative resection. Besides its well documented protective effect on the incidence of venous thromboembolism (VTE) in CRC surgical patients, preclinical studies have reported on its antineoplastic properties with promising results [1,2,3,4,5]. Heparin and derivatives have been suggested to inhibit angiogenesis and affect the progression of malignancy [6]. Tumor-mediated hematogenous metastasis has been postulated to be modulated by heparin derivatives by inhibiting the interaction between platelets and tumor cells and attenuating lymphatic metastasis by inhibiting lymphangiogensis [7].

Heparin is a mixture of glycosaminoglycans (GAGs) that are broadly classified into two types: (a) unfractionated heparin (UH) and (b) LMWH [7]. Many new derivatives have been synthetically created, some of which have also been explored for their anti-neoplastic properties [7]. Examples of these include low molecular weight heparins (LMWH) such as tinzaparin and deltaparin, synthetic heparin mimetics such as G2.2 and Tet-29, heparin sulfate interacting factors, and others [5,6,7,8,9,10,11,12,13,14,15,16,17,18,19,20].

The anticarcinogenic impact of heparin and derivatives has been investigated in a variety of study populations ranging from cell lines, tissues, and mice, to human patients in several types of malignancy [21,22]. A systematic review and meta-analysis of randomized trials by Lazo-Langer et al. on the antitumor effect of LMWHs demonstrated that it improves overall survival, even in patients with advanced malignancy [23]. However, there is still a lack of consensus on the anti-cancer properties of heparin and derivatives specifically for the CRC patient. In this context, we designed a study aiming to review the literature in order to determine the antineoplastic effect of heparin and heparin-stimulating substrates on CRC. This will allow for an improved understanding of the potential underlying anticarcinogenic effect that heparin may exert. In addition, we attempted to explore the mechanism under which heparin exerts its potential antineoplastic effects. 

## 2. Methods

### 2.1. Literature Search

This study was conducted in accordance with a protocol agreed upon by all of the authors and with the Preferred Items for Systematic Reviews and Meta-Analysis (PRISMA) guidelines [24]. A focused, systematic review of the literature was performed under the guidance of a qualified medical librarian to ensure a robust search strategy. MEDLINE (PubMed) and EMBASE databases were used to search for relevant articles. The last day of this search was the 11 August 2023. 

### 2.2. Search Strategy

Research articles from 1995 onwards were considered. A combination of MeSH terms and keywords were used to produce the search strategy. To identify studies reporting on antineoplastic mechanisms, we combined the MeSH terms “anticarcinogenic agents” and “venous thromboembolism” with the keywords “anticarcinogen”, “antiangiogenic”, “anticancer”, “thrombophylaxis”, and “venous thromboembolism”. To identify studies utilizing LMWH and other heparin derivatives the MeSH terms “LWMH” and “Heparin” were combined with the keywords “LMWH”, “Heparin”, and “Kallistatin”. Finally, to identify studies reporting on colorectal cancer the MeSH term “colorectal cancer” was combined with the keyword “colorectal cancer”. 

### 2.3. Citation Management and Data Extraction

Two independent reviewers performed title and abstract screening. Any disagreements were discussed and resolved with senior authors in group meetings. Full text review was performed by two authors independently. Studies that could not be retrieved were requested and provided by the medical library team. Data extraction was conducted independently by two authors with the following data points extracted from the included studies: first author, year of publication, primary study center, funding, conflict of interest, study design, study population, number of participants, type of heparin administered, dose, route of administration, adjunctive treatments, follow-up, method of CRC induction, method used to measure antineoplastic effect, results, and main finding. Extracted data were organized in a comprehensive Microsoft Excel spreadsheet (Version 16.76). 

### 2.4. Inclusion Criteria

Inclusion required the studies to report on the antineoplastic effects of heparin on colorectal cancer. In vivo and in vitro studies conducted on cells, animals and humans were included in this review. No minimum follow up was required for study inclusion. Studies utilizing LMWH, heparin derivatives and substrates that stimulate the heparin molecular pathway were included. 

### 2.5. Exclusion Criteria

Studies reporting only on the antithrombotic effects of heparin, without exploring any potential anticarcinogenic effects were excluded. Similarly, studies that examined the antineoplastic effect of heparin on other types of malignancy besides colorectal cancer were excluded. Individual case reports, abstracts, letters to the editor, book chapters and non-English articles were excluded. 

## 3. Results

**Figure 1:**
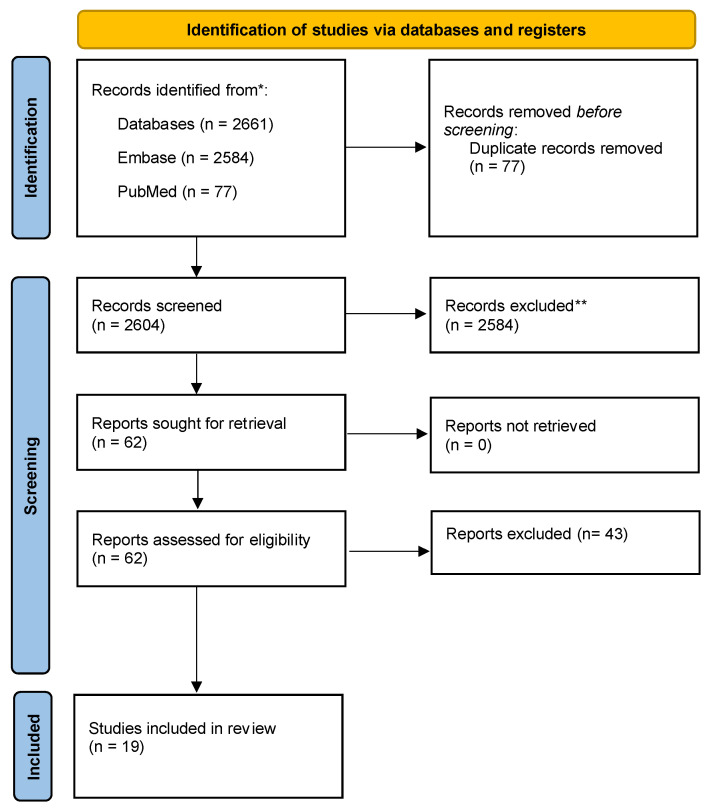
PRISMA flow diagram of included studies. * is to demonstrate the total studies included whereas the ** those excluded at title and abstract screening.

A total of 19 studies were included in this literature review (Figure 1). The reviewed articles were lab studies (*n* = 15), a retrospective cohort study (*n* = 1), a prospective cohort study (*n* = 1) and two randomized control trials (*n* = 2). 

### 3.1. In Vitro Cell Studies

Ten studies reported on outcomes of interest on cell lines in vitro (*n* = 10) [1,2,3,4,5,6,7,8,9,10]. Four of these articles explored the antineoplastic effects of heparin, though only at the cellular level in vitro lab studies (*n* = 4) [1,2,3,4]. Six studies explored similar outcomes in both in vitro cell lines and in vivo in rats (*n* = 6) [5,8,9,10,11,12]. The cell lines examined were human CRC cells (SW480, HT-29, HCT-116, HCT-199, LS-174, Caco-2, RKO, SW620) or murine CRC cell lines (CMT-93, MC38) [1,2,3,4,5,8,9,10,11,12]. The type of heparin that was administered included heparin sodium salt, LMWH, heparin surface interacting protein, and heparin stimulating molecules such as SERPINA3K (kallikrein-binding protein), kallistatin–LRP6 complex, midkine, SERPINA4 (kallistatin), YKL-40 and the heparin mimetics Tet-29 and G2.2 [1,2,3,4,5,8,9,10,11,12]. 

Anti-apoptotic effects were measured by apoptosis analysis using annexin V/propidium iodide stain or flow cytometric analysis of annexin V [2,3,4]. Cell viability was assessed using MTT assay while Western blots were employed to determine which proteins were upregulated or downregulated as a result of heparinergic substrates [2,3,4,5,8,9,10,11,12]. RNA (siRNA and mRNA) was used to evaluate gene activity, while cell cycle analysis was performed with DNA flow cytometry [2,3,4,8,9]. Cell growth, adhesion, migration, and invasion assays were also found to be commonly performed [1,5,8,9,10]. Additionally, parameters such as cell colony formation, overall cell survival and tumor growth were examined [5,8,11,12].

A significant reduction in cell viability was observed (33% reduction with SW480 and 53% with HCT116) in the heparin-treated groups [2,4,11,12]. Cell viability assays suggested that the inhibitory effect of heparin was dose dependent [3,4,8,11,12]. At the same time, cell lines with heparin-like substrates were found to have delayed tumor growth and improved overall survival [5,11,12]. The delayed tumor growth was established by assessing the number of colonies of tumor cells on soft agar or spheroid cultures. The reduction in tumor growth for both G2.2 and midkine were found to be dose dependent [5,11,12]. Furthermore, induced apoptosis was significantly higher when compared with non-treated cell lines, while LMWH inhibited cell colony formation and adhesion without a direct effect on proliferation [1,5,9,10]. Examination of mRNA levels of β-catenin, cyclin D1, and c-Myc revealed that they were significantly reduced in heparin-treated cell lines [2,8]. An increased proportion of cells in the heparin-treated cell lines were arrested in the G1/G0 cell cycle phase [2,8]. 

### 3.2. In Vivo Rat Studies

Eleven of the reviewed studies examined the anti-carcinogenic effect of heparin on rats (*n* = 11) [5,6,8,9,10,11,12,13,14,15,16]. A range of heparin and heparin-like molecules were used; unfractionated heparin (UFH), LMWH (reviparin, dalteparin, nadroparin, enoxaparin), kallistatin, LHD4 (derived from fraxiparine), midkine (heparin-binding growth factor), YKL-40 (heparin-binding glycoprotein), G2.2 and Tet-29 (both heparin mimetics) [5,6,8,9,10,11,12,13,14,15,16]. CRC carcinogenesis was induced in most of the studies by injecting the human or mural CRC cell lines mentioned earlier subcutaneously into the rats [5,6,8,10,11,12,13,15,16]. One study induced CRC by administrating mice azoxymethane (AOM) and dextran sodium sulfate (DSS), mimicking colitis-associated carcinogenesis [14]. Outcomes of interest included tumor growth and proliferation, microvessel density and/or maturation, distant metastasis (liver), anti-activated factor X (aXa), histopathological and immunohistochemical findings, and protein expression [5,6,7,8,9,10,11,12,13,14,15,16]. The follow up periods ranged from 8 days to 17 weeks. 

Tumor growth reduced by 78% (*p* < 0.01), while there was suppression of tumorigenesis in heparin-treated mice when compared with control groups [5,8,10,11,12,13,14]. A similar pattern was observed when examining polyp size, crypt hyperplasia and degree of tissue with neoplastic or premalignant changes [9,14]. With respect to microvessel density (MVD), two studies reported that heparin appeared to significantly reduce the absolute number of tumor vessels, 73 in experimental vs. 236 in control group (*p* < 0.01). MVD was 7.6% control vs. 5.5% nadroparin vs. 3.9% enoxaparin (*p* < 0.05) [13,15]. Similar findings have been reported with regards to degree of microvessel maturation, 77.9% control vs. 112.5% nadroparin and 106.5% enoxaparin (*p* = 0.05 and *p* = 0.034) [15]. However, one study examining the microvessel density of distant liver metastasis did not identify any significant difference between the LMWH, UFH and placebo treated mice [6]. 

Three studies focused on the effect of heparin on liver CRC metastasis, with two demonstrating that LMWH inhibited liver homogenate-induced cell migration and invasion (*p* < 0.05) [6,10,16]. LMWH was injected into mice with established CRC and the incidence of liver metastatic nodules was recorded [16]. LMWH-treated mice had a significantly smaller number of metastatic liver nodules (*p* < 0.05) [10]. The inhibition of liver-induced cell migration and invasion was also demonstrated by the decreased expression of CXCL12 (*p* < 0.05) on LMWH-treated mice [10]. All mice in the LMWH-treated group survived until 8 days, while only 75% of the control survived [16]. LMWH prevented metastatic tumor growth by 70% (69.14 × 10^3^ mm^3^ vs. 284.3 × 10^3^, *p* < 0.001) [16]. However, no difference in the colon cancer metastasis tumor volume was observed [6]. 

### 3.3. In Vivo Human Studies

Four studies investigated the antitumor effects of heparin in a total of 1000 human CRC patients [17,18,19,20]. Of these patients, 947 had biopsy-proven CRC and underwent curative (R0) resection [17,18,20]. The remaining 53 had advanced uncurable CRC malignancy [19]. Two of the in vivo human studies were cohort (retrospective and prospective) while the others were randomized control trials (RCT) [17,18,19,20]. The anti-tumor effect of LMWH (tinzaparin, fondaparinux, and nadroparin) was examined by measuring (1) its effect on VEGF levels following surgery, (2) recurrence-free survival, (3) mortality, and (4) safety [17,18,19,20]. The follow up periods ranged from 30 days up to 61.2 months. 

The effect of different doses (3500 IU or 4500 IU) and duration (10 days or 30 days) of subcutaneous tinzaparin revealed that patients who had a longer duration of administration had significantly lower VEGF levels at post-op day 30 compared with those with a shorter course of administration [17]. All patients, independent of the dose and duration of tinzaparin, had significantly higher VEGF levels on days 10 and 30 when compared with pre-operative levels [17]. Recurrence free survival in CRC patients who had a 4 day post-operative thrombophylaxis course with fondaparinux was the same as those who did not receive any LMWH at a median follow up of 47.8 months (FPX) and 61.2 months (control) [18]. The RCT by Klerk et al. 2005 demonstrated that 6 month survival in patients who received subcutaneous nadroparin for 4 weeks was 61% vs. 56% for placebo [19]. At 12 months survival this was 39% nadroparin, and 27% placebo, with a hazard ratio (HR) = 0.75 [95% CI, 0.59 to 0.96 (*p* = 0.021)] favoring the nadroparin group [19]. Disease-free survival occurred in 77% of the extended-duration tinzaparin group and in 79% of the in-hospital thrombophylaxis group, with an HR = 1.1 [95% CI, 0.90 to 1.33] [20].

Table 1 presents the study characteristics and main findings of the reviewed articles. This includes the name of the first author, year of publication, study design and population, type of heparin administered and the main findings. Heparin sulfate-interacting protein (HIP); kallistatin (KAL); vascular endothelial growth factor (VEGF); colorectal cancer (CRC); venous thromboembolism (VTE); intermittent pneumatic compression (IPC); fondaparinux (FPX).

Table 2 presents the colorectal cancer (CRC) molecular pathways that heparin and its derivatives may potentially affect. 

Mitogen-activated protein kinases (MAPK); C-X-C chemokine receptor 4 (CXCR4); C-X-C chemokine ligand 12 (CXCL12); vascular endothelial growth factor (VEGF); basic fibroblast growth factors (bFGF).

## 4. Discussion

The main finding of this literature review was that heparin and its derivatives attenuate CRC in cell lines and rat lab studies. This was demonstrated by the delayed tumor growth and improved overall survival of heparin-treated human and murine CRC cell lines, and by the suppressed tumorigenesis in mice. The anticarcinogenic effect of heparin in human clinical trials was less evident. Some clinical trials provided encouraging data on the role of heparin in the management of CRC, but others questioned whether it has any overall survival benefit. 

In vitro lab studies on human and murine CRC cell lines reported promising findings on the antineoplastic effect of heparin and its derivatives [1,2,3,4,5,8,9,10,11,12]. Heparin reduced CRC cell viability in a dose-dependent manner, while heparin derivatives delayed tumor growth and improved overall survival [2,3,4,5,8,11,12]. Heparin sodium salt and LMWH (enoxaparin) induced apoptosis in CRC cell lines and inhibited cell colony formation and adhesion [1,10]. This is an important finding because it suggests that heparin and LMWH may directly influence survival of CRC cancer cells and promote apoptosis [1,10]. Substrates such as kallistatin, a potent anti-angiogenic molecule which has a heparin binding site, demonstrated significant anti-tumor properties suppressing angiogenesis and retardation of colonic tumors [2,8,13]. This kallistatin–heparin interaction could therefore provide an interesting therapeutic target for CRC [2,8,13]. Cellular indicators of increased proliferation, such as mRNA levels of cyclin D1 and c-Myc, were significantly reduced in heparin-treated cell lines [2,8], indicating that heparin reduced the rate of CRC cell proliferation. This further reinforces the hypothesis that heparin has a direct anti-proliferative effect. 

Lab studies in rats have demonstrated that heparin and its derivatives suppressed CRC tumorigenesis [5,8,10,11,12,13,14]. This pattern has also been observed during histopathological examination of mice with CRC, as heparin-treated mice had significantly smaller sized polyps, reduced crypt hyperplasia and a smaller percentage of tissue with neoplastic or premalignant changes [9,14]. Overall, these findings strongly suggest that heparin and its derivatives had a direct impact on CRC growth and development in treated mice. 

Tumor microvessel density was significantly reduced in heparin-treated CRC mice (*p* < 0.01) [13,15]. Similarly, the degree of microvessel maturation was higher in nadroparin-and enoxaparin-treated mice [15]. These findings suggest that LMWH directly inhibited tumor progression and development on a microvascular level. Specifically, LMWH appeared to exert an anti-apoptotic effect on CRC slowing down angiogenesis and promoting healthy mature vessel development [13,15]. However, Smorenburg et al. did not identify any differences in metastatic vessel density in UFH- and LMWH-treated mice [6]. This may indicate that the anti-apoptotic effect of LMWH may potentially be more significant on the primary tumor, with limited effect once there is distant metastasis. 

Furthermore, LMWH attenuated CRC liver metastasis by inhibiting cell migration and invasion [10,16]. This was demonstrated by the smaller number of metastatic liver nodules in LMWH-treated mice and reduced cell migration and invasion [10,16]. Ma et al. have suggested that the mechanism via which LMWH reduces hepatic metastasis of colon cancer was its disrupting of the interaction between CXCR4 and CXCL12 [10]. However, Smorenburg et al., who compared the effects of heparin and unfractioned heparin on WAG-Rjj rats, reported no difference in the colon cancer metastasis tumor volume and metastatic vessel density on days 7 and 24 [6]. All three studies had similar study follow up periods, from 7 days to 24 days, and used a variety of human and mural CRC cell lines to induce CRC in the mice that participated in the experiment. The discrepancy of reported results is concerning, and hence further higher quality studies are needed to determine the anti-metastatic properties of LMWH on CRC in vivo rat studies.

The antineoplastic characteristics of heparin and its derivatives in human clinical trials were less evident. Four human trials were identified, two being RCTs and two cohort studies. Mitsis et al. examined the effect of LMWH on perioperative VEGF levels [17]. Anti-VEGF drugs, such as aflibercept, are increasingly being used in combination with chemotherapy to treat metastatic CRC, as the role of VEGF in promoting tumor angiogenesis has been extensively described in the literature [25,26]. Extended duration of tinzaparin resulted in significantly lower VEGF levels on post-op day 30 [17]. This finding demonstrates that administration of tinzaparin for an extended duration allowed for the normalization of VEGF within 30 days post-op in CRC patients. Therefore, due to its effect on VEGF, such regimes of LMWH may slow down CRC progression. However, a short-term post-operative LMWH thrombophylaxis course did not significantly affect VEGF or long-term disease-free survival [17,18]. Yamoaka et al. conducted a retrospective study comparing the disease-free survival of CRC patients who received a short 4 day LMWH thrombophylaxis course and intermittent pneumatic compression (IPC) with those who only had IPC. No difference in disease-free survival between the two groups at a median follow up of 47.8 months (about 4 years) and 61.2 months (about 5 years), respectively, was observed [18]. Overall, the data demonstrate that a short course of LMWH does not influence CRC disease progression. However, a high-dose, extended-duration LMWH regime could inhibit CRC progression and distant metastasis by normalizing VEGF levels faster following surgery. 

Two RCTs with confounding results were included in this review [19,20]. Klerk et al. explored the overall survival in patients with uncurable advanced solid malignancy, with 53 of the included 302 participants having CRC [19]. Twelve month survival was 39% in the nadroparin group, and 27% in the placebo group, with a hazard ratio (HR) of 0.75 [95% CI, 0.59 to 0.96 (*p* = 0.021)] favoring the nadroparin group [19]. However, they did not perform a subgroup analysis investigating survival specifically the CRC cohort. Auer et al. in 2022 explored the effect of an extended duration of tinzaparin on disease-free survival (DFS) in 614 adults undergoing CRC surgical resection. They demonstrated that DFS occurred in 77% in the extended duration tinzaparin group and in 79% in the in-hospital thrombophylaxis group, with an HR = 1.1 [95% CI, 0.90 to 1.33] [20]. The trial was stopped prematurely following interim analysis as it was becoming futile [20]. Both studies used LMWH with similar follow up and duration of administration. Overall data from the two RCTs reviewed were non-consistent.

The role of heparin as an adjuvant component of gene therapy for CRC has been previously reported in the literature [27,28]. Heparin–polyethyleneimine (HPE) nanoparticles and nanogels have been used as nonviral gene vectors for a safe and efficient delivery of gene therapy for colon cancer in vitro and in vivo. HPEI successfully transferred the gene therapy efficiently, inducing apoptosis and inhibiting angiogenesis while at the same time inhibiting the growth of pulmonary metastasis [27,28]. These studies have demonstrated the promising role of heparin-coated nanoparticles as a delivery system of gene therapy for CRC. 

One of the most common postoperative complications following CRC resection are surgical site infections (SSIs), which contribute to significant perioperative morbidity, and extended length of hospital stay [29]. SSIs have also been associated with a negative economic impact [29]. However, use of prophylactic unfractionated heparin does not appear to affect the incidence of SSI in patients undergoing colorectal surgery [30]. In the future, the development of machine learning models may have the capacity to quantify the risk of heparin administration in surgical CRC patients [31]. Interestingly, some studies have recently described the role of heparin as a stable carbon source for the gut microbiota [32].

The results of this study must be interpreted in the context of its limitations. Firstly, a wide range of study populations were reported in the literature, making it difficult to compare groups and results between different studies. In particular, the review included in vitro and in vivo studies with a range of study populations and outcomes. Furthermore, as discussed earlier, Klerk et al. have reported on 302 patients with advanced solid malignancy, of whom 53 had CRC. Unfortunately, no subgroup analysis was performed on the 12 month survival for each malignancy type. Therefore, we have presented their total findings for overall survival and HR, which may have underestimated or overestimated the effect of LMWH on overall survival. 

Future high-quality randomized control studies investigating the antineoplastic effect of heparin are needed to determine the clinical relevance of the findings observed in in vitro and in vivo rat studies. These studies should also explore different doses and duration of administration of heparin and its derivatives in order to determine which regime could be associated with improved outcomes. 

## 5. Conclusions

Heparin and its derivatives appear to have an antineoplastic effect on CRC cell lines and in rat lab studies. However, this effect was less evident in human clinical studies. Heparin may provide an important additional treatment option for CRC patients due to its promising findings in lab studies and safe profile in clinical studies. However, future studies comparing different doses and duration of administration are needed to explore which regimes may indeed have a clinical benefit on disease-free survival in CRC patients. 

## Figures and Tables

**Table 1:** Study characteristics and main findings of reviewed articles.

Study Number	Author	Primary Study Centre	Study Design	Study Population	Study Groups	Type of Heparin Administered	Main Finding
1	Antachopoulos 1995 [1]	Greece	In vitro, lab study	Human colon adenocarcinoma cells (SW480)	Heparin groups with different concentrations and control	Heparin sodium salt	Chemically modified heparins probably play a role in the in vivo inhibition of tumor cell metastasis
2	Shahbazi 2023 [2]	Iran	In vitro, lab study	Human CRC cells (SW480 HCT116)	Kallistatin and control	Kallistatin–LRP6 complex	Kallistatin has anti-tumor effects
3	Liu 2004 [3]	Singapore and USA	In vitro, lab study	Human CC Cell Lines (HT-29 and HCT-116)	Sodium butyrate, adherent and floating cell lines	Heparin sulfate-interacting protein (HIP)	HIP is an anti-apoptotic peptide and is involved in regulating apoptosis induced by anticancer drugs
4	Yao 2013 [4]	China	In vitro, lab study	Human CRC cells (SW480 and HT-29)	SERPINA3K and control	SERPINA3K (kallikrein-binding protein)	SERPINA3K exerted anti-tumor activity by suppressing the rate of proliferation and inducing CRC apoptosis
5	Takei 2001 [5]	Japan	In vitro and in vivo, lab study	Mice rectal carcinoma cells (CMT-93), athymic nude mice from SLC (Tokyo, Japan)	NR	Midkine (heparin-binding growth factor)	Inhibitory effect of midkine antisense oligodeoxynucleotide on rectal carcinoma
6	Sun 2016 [8]	China	In vitro and in vivo, lab study	Fresh primary cancer specimens and matched normal mucosa Human CRC cell lines (HCT116, RKO, SW620, SW480, Caco2, LoVo, HT29, and HCT8) Male BALB/c mice	CRC mucosa matched with healthy colonic mucosa Cell cultures Mouse xenografts	SERPINA4 (kallistatin)	SERPINA4 is significantly correlated with aggressive phenotype and poor clinical outcomes in CRC. SERPINA4 suppresses cancer progression and serves as a potential therapeutic target for CRC
7	DeRobertis 2022 [9]	Italy and Bulgaria	In vitro and in vivo, lab study	Human CRC cell lines (9HCT116 and Caco2), BALB/c female mice, and 41 paired CRC mucosa and healthy mucosa of human patients	NR	YKL-40 (heparin-binding glycoprotein)	YKL-40 appeared to promote the metastatic phenotype during CRC carcinogenesis
8	Ma 2012 [10]	China	In vitro and in vivo, lab study	Male nude BALB/c mice and human colon cancer cell lines (HT29, LS-174 T, HCT-199)	Control, CXCL12, CXCL12 and LMWH, CXCL12 and CXCR4 Ab, Placebo, LMWH	LMWH (enoxaparin)	LMWH may help prevent the seeding and subsequent growth of hepatic metastasis of colon cancer cells
9	Boothello 2019 [11]	USA	In vitro and in vivo, lab study	Human CRC cells (HT29 and HCT116) and pancreatic (Pan = 1), female NCr nude mice	NR	G2.2 (mimetic of heparin hexasaccharide)	G2.2 is a promising therapeutic agent for cancer
10	Spijkers-Shaw 2022 [12]	New Zealand	In vitro and in vivo, lab study	MC38 murine model of colon adenocarcinoma were implanted in flanks of syngeneic C57BL/6 mice, HT29 human colorectal adenocarcinoma cells	Treatment, control	Tet-29 (heparin sulfate mimetic developed in the lab)	Novel glycolipid (HS mimetic) delayed tumor growth and improved overall survival
11	Diao 2007 [13]	China	In vivo, lab study	Male BALB/c mice	rAVV-GFP, rAVV-KAL	Kallistatin (KAL)	KAL suppressed angiogenesis and resulted in growth retardation of colon tumors
12	Smorenburg 1999 [6]	Netherlands	In vivo, lab study	WAG-Rjj Rats	LMWH, UFH, control	LMWH (reviparin), UFH	Heparins do not affect colon carcinoma metastasis in liver
13	Kim 2014 [14]	South Korea	In vivo, lab study	Male ICR mice	Control, AOM and DS, celecoxib, LHD4, celecoxib and LHD4	LHD4 (derived from fraxiparine, 4500 Da)	The combined use of celecoxib and LHD4 could significantly enhance chemoprevention of CRC in terms of polyp formation and malignancy development
14	Debergh 2015 [15]	Belgium	In vivo, lab study	Male athymic mice with human CC cell line (HT29)	Group I—0.1 mL saline Group II—200 IU aXa nadroparin Group III—200 IU aXa dalteparin	Nadroparin, dalteparin	Nadroparin and enoxaparin inhibit tumor-associated angiogenesis and normalize microvessel structure in this mouse xenograft
15	Djaafar 2016 [16]	Switzerland	In vivo, lab study	Mice wild type C57BL/6 with mouse CC cells (MCA38) and mouse melanoma cells (B16-F10 BL6)	Enoxaparin and placebo (phosphate buffered saline)	Enoxaparin	Enoxaparin significantly reduced CC metastatic tumors in the mouse liver at early stages of development.
16	Mitsis 2017 [17]	Greece	In vivo, prospective cohort study	Human patients with endoscopy-biopsy-proven CC undergoing colectomy with curative intent	Group I: 3500 I.U. for 10 days, Group II: 3500 I.U. for 30 days, Group III: 4500 I.U. for 10 days, Group IV: 4500 I.U. for 30 days	LMWH (tinzaparin)	Post-op VEGF levels may contribute to future progress of disease. The use of high-dose tinzaparin for a long period may help better control VEGF fluctuations.
17	Yamaoka 2016 [18]	Japan	In vivo, retrospective cohort study	Human with primary CRC and pathologically diagnosed lymph-node metastasis who underwent curative (R0) resection	Fondaparinux (FPX) and intermittent pneumatic compression (IPC) vs. intermittent pneumatic compression (IPC) only	Fondaparinux (FPX)	Short-term postoperative use of FPX as VTE prophylaxis does not prevent CRC recurrence after curative resection.
18	Klerk 2005 [19]	Netherlands	In vivo, randomized control trial	Human patients with uncurable advanced solid organ malignancy	Nadroparin and placebo	Nadroparin	Six week course of LMWH in patients with advanced solid malignancy reduces mortality at 12 and 24 months by 12% and 10% and prolongs median survival from 6.6 months to 8.0 months
19	Auer 2022 [20]	Canada	In vivo, randomized control trial	Patients with CRC, no evidence of metastatic disease, and scheduled to undergo surgical resection	Treatment and control	LMWH (tinzaparin)	Extended duration to perioperative thrombophylaxis with tinzaparin (given before surgery and for 56 days after surgery) does not increase disease-free survival at 3 years. Rates of clinically detected VTE were low and extended-duration thrombophylaxis was not associated with a reduction in VTE.

**Table 2:** CRC molecular pathways that heparin and its derivatives may potentially affect.

Molecular Pathway	Mechanism
p38 MAPK	Activation of p38 MAPK to inhibit CRC [9,11]
Wnt signaling pathway	Inhibited [2]
β-catenin	Reduced expression in heparin treated cells and rats [2]
Cyclin D1	Reduced expression in heparin treated cells and rats [2]
c-Myc	Reduced expression in heparin treated cells and rats [2]
Caspase 3	Activation [3]
FasL/caspase-8	Activation [4]
PI3K/AKT signaling pathway	Activation [9]
CXCR4-CXCL12	Downregulates [10]
VEGF and bFGF	Reduce VEGF and bFGF binding activity [13,15,17]

## Data Availability

Data are contained within the article.
